# Presence of Streptococcus dentisani in the dental plaque of children from different Colombian cities

**DOI:** 10.1002/cre2.158

**Published:** 2019-05-09

**Authors:** María P. Angarita‐Díaz, Jaime A. Díaz, Herlinto A. Tupaz, Arantxa López‐López, Diana Forero, Alex Mira, Fernando Dávila, Ximena A. Cerón, Emilia M. Ochoa‐Acosta, Olga L. Goméz, Gladys Gonzalez

**Affiliations:** ^1^ School of Dentistry Universidad Cooperativa de Colombia, Villavicencio Campus Villavicencio Colombia; ^2^ School of Dentistry Universidad Cooperativa de Colombia, Bogotá Campus Bogotá Colombia; ^3^ Department of Health and Genomics Foundation for the Promotion of Health and Biomedical Research, FISABIO Valencia Spain; ^4^ School of Dentistry Universidad Cooperativa de Colombia, Pasto campus Pasto Colombia; ^5^ School of Dentistry Universidad Cooperativa de Colombia, Medellín campus Medellín Colombia; ^6^ School of Dentistry Pontifica Universidad Javeriana Bogotá Colombia

**Keywords:** bacteria, child, dental caries, probiotics, real‐time PCR

## Abstract

Streptococcus dentisani has been identified as an oral cavity probiotic due to its beneficial characteristics. One of its beneficial features is the production of bacteriocins, which inhibit the growth of cariogenic bacteria, and another is its buffering capacity through the production of ammonium from arginine. The purpose of this study was to determine the presence of S. dentisani in the dental plaque of Colombian children and whether the presence of this bacterium is related to oral health and other conditions. Dental plaque and information on diet and oral hygiene habits were collected from children between 6 and 12 years of age from four Colombian cities, divided into caries‐free children (International Caries Detection and Assessment System [ICDAS] 0, Decayed Missing Filled Teeth index [DMFT] 0), children with ICDAS 1 and 2, and children with ICDAS >3. Plaque DNA was extracted and quantified, and real‐time polymerase chain reaction was performed using specific primers. This bacterium was identified in all samples, with a median of 0.46 cells/ng DNA (interquartile range [IQR] 0.13–1.02), without finding significant differences between the groups (*P* > 0.05). In caries‐free children, a median of 0.45 cells/ng DNA (IQR 0.14–1.23) was found. In children with ICDAS 1 and 2, the median was 0.49 cells/ng DNA (IQR 0.11–0.97), and in children with ICDAS >3, the median was 0.35 cells/ng DNA (IQR 0.12–1.07). However, statistically significant differences were found in the origin of children (*P* < 0.01), the use of fluoride‐containing products (*P* < 0.01), and the frequency of food intake (*P* < 0.05). In conclusion, the presence of S. dentisani was quantified in children from four Colombian cities, without finding significant differences in oral health status. Nevertheless, three conditions showed a possible relationship with S. dentisani.

## 1 INTRODUCTION

The oral cavity includes several niches, such as the surfaces of teeth and cheeks, periodontal pockets, tongue, saliva, gingival sulcus, and soft and hard palates, among others. Each region of the mouth has its own characteristics, with unique microenvironments that allow the establishment of the oral microbiome, where bacteria predominate with over 700 different species (Kilian et al., 2016). The literature describes the composition of the oral microbiome, analyzes its roles in healthy and unhealthy mouths, and infers the interactions between the oral microbiome and its host (Jiang, Gao, Jin, & Lo, 2016; Kilian et al., 2016; Simón‐Soro, & Mira, 2015; Zaura, Nicu, Krom, & Keijser, 2014).

After birth, a newborn acquires a wide variety of microorganisms. Only part of them are able to colonize the individual, which can influence the subsequent colonization by other microorganisms (Sampaio‐Maia & Monteiro‐Silva, 2014). The initial colonization process is dominated by *Streptococcus*, which make up to 80% of microorganisms of the biofilm (Kreth, Merritt, & Qi, 2009), followed by *Actinomyces* and other bacteria. During maturation of the microbiome, environmental conditions, such as oxygen concentration, pH, diet, salivary flow, hormonal changes, and oral hygiene habits, among others, modify the microbial community (Jiang, Gao, Jin, & Lo, 2016; Kilian et al., 2016; Sampaio‐Maia & Monteiro‐Silva, 2014; Simón‐Soro, & Mira, 2015; Zaura, Nicu, Krom, & Keijser, 2014).

Advances in molecular biology have allowed the development of methods favoring the knowledge of the diversity and composition of the oral microbiome, the understanding of the dynamics and establishment of microorganisms, and their roles in health and disease (Benn, Heng, Broadbent, & Thomson, 2018). Real‐time quantitative polymerase chain reaction (qPCR) allows simultaneous amplification and quantification of the amplicons using a fluorescent dye. This dye or fluorophore binds only to double‐stranded DNA after each amplification cycle; therefore, the fluorescence intensity reflects the number of DNA amplicons generated. The point at which fluorescence intensity increases above the detection threshold corresponds proportionally to the initial number of molecules of the sample DNA template. This point is called the quantification cycle (Cq) and allows the absolute amount of target DNA to be determined according to the constructed calibration curve. This technique is highly accurate and sensitive for the quantification of individual bacterial species as long as appropriate and specific primers are used (Kralik P, & Ricchi, 2017).

Different bacterial species are associated with mouth diseases, such as Streptococcus mutans, *Lactobacillus* (Becker, Paster, & Leys, 2002) and Scardovia wiggsiae (Kressirer et al., 2017) in caries; Porphyromonas gingivalis, Treponema denticola, Tannerella forsythia (Mineoka et al., 2008), Prevotella intermedia, and Prevotella nigrescens (Zhang et al., 2017) in periodontal disease; or Fusobacterium nucleatum in halitosis (Lee, Mak, & Newsome, 2004). In contrast, healthy mouth conditions are associated with other species with interesting characteristics to control the dysbiosis. Streptococcus salivarius *K12* (Burton et al., 2013), the *Streptococcus* strain designated A12 (Huang et al., 2016), and Streptococcus dentisani (López‐López, Camelo‐Castillo, Ferrer, Simon‐Soro, & Mira, 2017) are some examples of the beneficial bacteria within the oral cavity that inhibit the pathobionts, restoring the microbial ecological balance. In Colombia, there are no reports available on the determination of beneficial bacteria with oral probiotic characteristics and the possible host conditions that determine their presence.

S. dentisani belongs to the Mitis group and has been isolated from the dental plaque of caries‐free Spanish individuals (Camelo‐Castillo, Benítez‐Páez, Belda‐Ferre, Cabrera‐Rubio, & Mira, 2014). This coccus‐shaped bacterium grows in colonies of approximately 1.5 mm in diameter, is a facultative anaerobe, and has an optimum pH of 7, although it resists moderately acidic conditions (Camelo‐Castillo, Benítez‐Páez, Belda‐Ferre, Cabrera‐Rubio, & Mira, 2014). One of its beneficial features is the production of bacteriocins, which inhibit the growth of cariogenic bacteria, such as S. mutans and Streptococcus sobrinus; another is its buffering capacity through the production of ammonium from arginine (López‐López, Camelo‐Castillo, Ferrer, Simon‐Soro, & Mira, 2017). Consequently, Spanish researchers have proposed this bacterium as a probiotic to promote oral health. The purpose of this pilot study is to determine the presence of S. dentisani in the dental plaque of children from different Colombian cities (Bogotá, Medellín, Pasto, and Villavicencio) and whether the presence of this bacterium is related to oral health and other conditions, such as origin, oral hygiene, food and carbohydrate intake, or the use of fluoride products.

## 2 MATERIALS AND METHODS

This exploratory study involved a convenience sample including 100 children divided into the following groups: caries‐free children (without the presence of caries and restorations International Caries Detection and Assessment System [ICDAS] 0 or Decayed Missing Filled Teeth index [DMFT] 0), children with ICDAS 1 or 2 (early caries), and children with ICDAS >3 (moderate and extensive caries). Samples were collected in four different cities of Colombia (Table 1). The inclusion criterion was an age between 6 and 12 years, and the exclusion criteria were having basic systemic pathologies, having received antibiotics in the last 3 months, and having teeth brushed at least 8 hr prior to sample collection. Before starting the study, informed consent was requested from the parents of the children and also a questionnaire that requested personal information (geographic origin, sex, and age), diet information (frequency of food intake per day and frequency of fermentable carbohydrates intake per day), and oral hygiene habits (brushing frequency and use of fluoride‐containing products). This study was approved by the ethics subcommittee of the Universidad Cooperativa de Colombia, Villavicencio (21102015).

**Table 1:** Characteristics of the cities where samples were collected

City	Location	Altitude (masl)	Average temperature (°C)
Bogotá, capital of Colombia and of Cundinamarca Department	Central Colombia in the Eastern of the Andes Mountains	2,625	14
Pasto, capital of Nariño Department	Southwest of Colombia in the center of the Andes Mountains	2,527	12
Medellín, capital of Antioquia Department	Northwestern Colombia in the center of the Andes Mountains	1,479	24
Villavicencio, capital of Meta Department	Eastern central Colombia in the foothills of the Eastern Andes of Colombia	467	27

*Note*. masl: meters above sea level. Source: Official City Website.

### 2.1 International Caries Detection and Assessment System

For the diagnosis and selection of children, the ICDAS was used to determine carious activity. The dentists who made the diagnosis and collected the samples were calibrated in the ICDAS system, obtaining satisfactory inter‐ and extra‐examiner reproducibility (Kappa value ≥0.7).

### 2.2 Sample collection

Prior to sample collection, the dental plaque index was determined using the modified Silness and Löe index (Mombelli, Van Oosten, Schürch, & Lang, 1987). Subsequently, supragingival plaque was collected with a sterile curette, scraping over tooth surfaces (buccal, lingual, and occlusal) in Quadrants 1 and 3 for temporary and permanent dentition, without touching the gums. Samples were collected in microtubes containing 200 μl of sterile saline solution, to which the corresponding code was assigned. The samples were kept frozen at −20°C until molecular analysis.

### 2.3 DNA extraction and qPCR

For DNA extraction, 200 μl of each sample was used, to which 5 μl of lysozyme from chicken egg white (10 mg/ml, Sigma‐Aldrich, Dorset, UK). The samples were then incubated at 37°C for half an hour, after which the extraction was continued according to the protocol of the High Pure PCR Template Preparation Kit (Roche Diagnostics GmbH, Mannheim, Baden‐Württemberg, Germany), following the manufacturer's recommendations. DNA was quantified with a Nanodrop 2000c spectrophotometer (Thermo Scientific, Wilmington, Delaware, USA), and samples with an adequate quantity and quality of DNA were selected for further analyses. For qPCR, the CFX96 Touch™ Real‐Time PCR Detection System (Bio‐Rad, Berkely, California, USA) was used with the FastStart Universal SYBR Green Master (Rox) Kit (Roche Diagnostics GmbH, Mannheim, Baden‐Württemberg, Germany) using specific primers for S. dentisani, with a 25‐μl reaction volume. The specific primers used match with the gene encoding carbamate kinase (CkSdF5′‐GTAACCAACCGCCCAGAAGG‐3′ and CkSdR5′‐CCGCTTTCGGACTCGATCA‐3′; Mombelli, Van Oosten, Schürch, & Lang, 1987). Primers specificity was confirmed by the absence of amplification by qPCR from S. mutans, S. sobrinus, Streptococcus sanguinis, S. salivarius, Streptococcus mitis, Streptococcus infantis, and Streptococcus oralis (López‐López, Camelo‐Castillo, Ferrer, Simon‐Soro, & Mira, 2017). DNA of the reference strain of S. dentisani 7446 was used as a positive control, and water was used as a negative control to detect potential contamination. Prior to quantification, the technique was standardized, and the calibration curve was constructed according to López‐López et al. (2017). The concentration of S. dentisani in each sample was calculated by comparing the Cq value obtained to the standard curve. This curve was generated using serial dilutions of DNA extracted from the reference strain S. dentisani 7746. The Cq value obtained from dental plaque samples was replaced into the standard equation and expressed as an absolute number. The samples were normalized to determine the number of S. dentisani cells/ng of DNA present in the sample.

### 2.4 Statistical analysis

For statistical analyses, SPSS 25.0 software was used. To determine whether there were significant differences between the groups of each variable studied, and according to the normality of the data, nonparametric tests were performed, including the Mann–Whitney U (sex, use of fluoride‐containing products, and frequency of food intake) and the Kruskal–Wallis H tests (oral health status of children [ICDAS], origin, age, plaque index, brushing frequency, and fermentable carbohydrate intake per day).

## 3 RESULTS

One hundred dental plaque samples from children divided into three groups were evaluated: ICDAS 0 with 36 samples analyzed, ICDAS 1 and 2 with 32 samples, and ICDAS >3 with 32 samples. Table 2 shows the distribution according to each characteristic evaluated.

**Table 2:** Characteristics of the children in the study

Characteristic		*N* (%)
ICDAS	0	36 (36)
	1 and 2	32 (32)
	>3	32 (32)
Sex	Male	41 (41)
	Female	59 (59)
Origin	Bogotá	25 (25)
	Medellín	28 (28)
	Pasto	27 (27)
	Villavicencio	20 (20)
Age[Link]	6–8 years	68 (68)
	9–10 years	23 (23)
	11–12 years	9 (9)
Plaque index	Good	8 (8)
	Average	27 (27)
	Poor	65 (65)
Brushing frequency	Once a day	5 (5)
	2 times a day	27 (27)
	3 times a day	38 (38)
	4 times a day	30 (30)
Use of fluoride‐containing products	Yes	78 (78)
	No	22 (22)
Frequency of food intake per day	1–3	24 (24)
	>4	76 (76)
Frequency of fermentable carbohydrate intake per day	0	34 (34)
	1–2	17 (17)
	>3	49 (49)

Grouped by age according to stages of mixed dentition.

### 3.1 Identification and quantification of S. dentisani in dental plaque samples

All analyzed samples were positive for S. dentisani, with a median of 0.46 cells/ng DNA (interquartile range [IQR] 0.13–1.02), a minimum value of 0.001 cells/ng DNA, and a maximum of 7.20 cells/ng DNA. In caries‐free children, a median of 0.45 cells/ng DNA (IQR 0.14–1.23) was found, with minimum and maximum values of 0.002 and 7.20, respectively. In children with ICDAS 1 and 2, the median was 0.49 cells/ng DNA (IQR 0.11–0.97), with a minimum value of 0.003 and a maximum of 6.39; and in children with ICDAS >3, the median was 0.35 cells/ng DNA (IQR 0.12–1.07), with minimum and maximum values of 0.01 and 2.29, respectively. No statistically significant differences were found between caries‐free children and those with caries (Table 3).

**Table 3:** Analysis of the quantification of Streptococcus dentisani, with respect to the characteristics studied

Characteristic	Median	IQR	Range (minimum‐maximum)	*P*‐value
ICDAS				
0	0.45	0.14–1.23	0.002–7.20	0.98^c^
1 and 2	0.49	0.11–0.97	0.003–6.39	
>3	0.35	0.12–1.07	0.001–2.29	
Oral condition				
Caries‐free	0.45	0.14–1.23	0.002–7.20	0.91^d^
With caries	0.46	0.12–0.99	0.003–6.39	
Sex				
Male	0.42	(0.13–1.23)	0.001–6.39	0.95^d^
Female	0.47	(0.13–0.95)	0.003–7.20	
Origin				
Bogotá	0.69	0.33–1.16	0.05–2.29	0.00^b^^,^ c
Medellín	0.43	0.17–0.89	0.005–7.20	
Pasto	1.04	0.23–1.76	0.001–6.39	
Villavicencio	0.11	0.007–0.30	0.001–0.72	
Age^a^				
6–8 years	0.35	0.14–1.03	0.003–6.39	0.27^c^
9–10 years	0.88	0.13–1.23	0.002–7.20	
11–12 years	0.40	0.081–7.23	0.002–0.95	
Plaque index				
Good	0.52	0.13–0.87	0.08–1.63	0.34^c^
Average	0.28	0.05–0.99	0.003–1.75	
Poor	0.58	0.15–1.06	0.003–7.20	
Brushing frequency				
Once a day	*0.63*	0.1–0.97	0.08–1.08	0.07^c^
2 times a day	0.22	0.05–0.72	0.001–4.17	
3 or more times a day	0.67	0.18–1.26	0.005–6.39	
Use of fluoride‐containing products				
Yes	0.38	0.11–0.90	0.002–7.20	0.007^b^^,^ d
No	1.12	0.25–1.77	0.03–6.39	
Frequency of food intake per day				
1–3	1.04	0.19–1.72	0.009–6.39	0.039^b^^,^ d
>3	0.40	0.12–0.89	0.002–7.20	
Frequency of fermentable carbohydrate intake per day				
0	0.10	0.035–1.57	0.003–1.63	0.30^c^
1–2	0.57	0.26–1.13	0.004–7.20	
>3	0.40	0.13–0.98	0.002–2.29	

a Age‐grouped according to stages of mixed dentition.

b According to the statistical analysis, these groups showed significant differences (*P* < 0.05).

c Kruskal‐Wallis test.

d Mann‐Whitney U test.

When comparing the quantification of S. dentisani according to the groups related to characteristics such as sex, origin, age, plaque index, oral hygiene, and eating habits, statistically significant differences were found in the origin of children (*P* < 0.01), the use of fluoride‐containing products (*P* < 0.01), and the frequency of food intake (*P* < 0.05). Samples from children from Bogotá, Pasto, and Medellín had medians of 0.69 cells/ng DNA (IQR 0.33–1.16), 1.04 cells/ng DNA (IQR 0.23–1.76), and 0.43 cells/ng DNA (IQR 0.17–0.89), respectively; the median of the Villavicencio samples was 0.11 cells/ng DNA (IQR 0.007–0.30).

Children whose parents did not report the use of fluoride products had a significantly higher median of S. dentisani (1.12 cells/ng DNA, IQR 0.03–6.39) than those that used these products regularly (0.38 cells/ng DNA, IQR 0.002–7.20). Similarly, children who had a lower food intake (Jiang et al., 2016; Kilian et al., 2016; Zaura, Nicu, Krom, & Keijser, 2014) presented higher levels than those with higher intakes (1.04 cells/ng DNA, IQR 0.009–6.39 vs. 0.40 cells/ng DNA, IQR 0.002–7.20, respectively; Table 3).

## 4 DISCUSSION

In the present pilot study, S. dentisani was detected in the dental plaque samples of all the children evaluated, with a median of 0.46 cells/ng DNA (IQR 0.13–1.02), without finding significant differences between caries‐free children and those with some caries index level. One of the possible reasons for not finding a significant difference between the two groups could be the sample size and the large inter‐individual variation in bacterial load. It should also be noted that there are other factors involved in the presence and density of microorganisms.

One of these factors is the great diversity of microorganisms and interactions involved in disease or health processes, where it is not only one or a few microorganisms generating one state or the other. Currently, with advances in molecular techniques, it has been possible to identify many microorganisms involved in different processes, such as caries. Simón‐Soro, Guillen‐Navarro, and Mira (2014) used transcriptomics to identify the bacterial composition in caries lesions in different individuals (*n* = 13) and stages of disease progression. The authors found that the microbiota of carious lesions is highly complex because it is polymicrobial and because it is organized in consortiums that vary between individuals and even between caries lesions (Simón‐Soro, Guillen‐Navarro, and Mira 2014.

Similarly, other studies have shown that under healthy conditions, many bacteria are involved in beneficial processes, such as the production of ammonium for pH stabilization. Nascimiento et al., (2009), when using qPCR to quantify alkaline‐producing bacteria from arginine (S. sanguinis and Streptococcus gordonii) and from urea (S. salivarius and Actinomyces naeslundii), in patients with caries and caries‐free patients, found no significant differences in the proportions of S. sanguinis, S. gordonii, and A. naeslundii between the two groups, despite having found a higher ammonium production in the dental plaque of caries‐free patients. These findings suggested the presence and intervention of other ammonium‐producing bacteria that contributed to the process (Nascimiento, Gordan, Garvan, Browngardt, & Burne RA, 2009).

Another factor that could have conditioned the results of this study involves the particular physicochemical conditions of each habitat in the oral cavity interacting with the characteristics of each host, such as diet and oral hygiene habits. In this study, significant variations were detected in the quantification of S. dentisani among individuals of the same group. The quantification range in caries‐free children was 0.002–7.20 cells/ng DNA, whereas that of children with caries was 0.003–6.39 cells/ng DNA. Wake et al. 2016, in an in situ study on dental biofilm formation at different time intervals, demonstrated the fundamental roles of certain conditions, such as oxygen pressure, nutrient supplements, and pH in microbial diversity and quantification (Wake et al., 2016).

Therefore, the lifestyle can influence in the concentration of S. dentisani in the children. In this study, one condition that significantly influenced the quantification of S. dentisani is the amount of daily food intake. Children with a maximum frequency of food intake of 3 showed significantly higher quantification of S. dentisani than children with higher food intake. This difference could occur due to changes in the oral environment after food intake, resulting in differences in the multiplication and permanence of S. dentisani. One example would be the decrease in salivary pH. Because the optimum pH range for this bacterium is between 7 and 7.5, its growth would be expected to decrease as conditions reached an acidic pH. However, in this study, no significant quantification differences in S. dentisani were detected in children with higher or lower frequencies of fermentable carbohydrate intake, which are the type of carbohydrates that have the greatest effect in terms of pH reduction; however, it has been shown that a high dietary content of starches or fruits also reduces plaque pH (Moynihan, 2005) and the repeated acidic challenge imposed by multiple meals has been proposed to represent an important selective pressure against nonacidophilic microorganisms (Rosier, Marsh, & Mira, 2018).

Another aspect that may influence the quantification of S. dentisani in this variable is that after food intake, some microorganisms are metabolically activated, and through different interactions during biofilm formation (Benítez‐Páez, Belda‐Ferre, Simón‐Soro, & Mira, 2014), they may influence the presence of S. dentisani. This influence is mediated through the competition of nutrients and/or space or by the production of substances toxic to bacteria, such as bacteriocins.

This finding could indicate that diet is influencing the presence of this bacterium and, therefore, the relationship with the children's place of origin, because the altitude of the city (Villavicencio) that was home to the children with the lowest levels of S. dentisani is very different from those of the other cities. Altitude can determine the type of food that is grown and therefore more frequently consumed. This possible relationship with diet could be influenced by the endogenous nutritional environment (saliva, tissues, crevicular fluid, microbial metabolites, etc.) through systemic circulation. In an exploratory study involving metagenomic sequencing of 16S ribosomal RNA, Kato et al. (2017) reported an association between intake of a specific nutrient (saturated fat acids, vitamin C, and glycemic load) and microbial diversity. Saturated acid was correlated with relative abundance of Betaproteobacteria and Fusobacteria, vitamin C exhibited positive correlations with abundance in fusobacteria class and Leptotrichiaceae and Lachnospiraceae families, and finally, the glycemic load was positively correlated with Lactobacillaceae abundance (Kato, Vasquez, & Moyerbrailean, 2017).

In Africa, an important difference was detected on salivary microbiome composition between agricultural groups from Sierra Leona and Congo, and a former hunter‐gatherer group from Uganda (Batwa). This difference could be explained by the protein‐rich diet of the Batwa, being approximately half of hunted animal meat, in contrast to the predominantly agriculture diet of both Sierra Leona and Congo, which have similar lifestyle and diet although they are geographically distant (Nasidze, Li, Schroeder, Creasey, Li, & Stoneking, 2011). In addition to diet, other cultural condition such as hygiene and environmental exposure may influence in geography variation, but studies are required in oral microbiome.

Finally, children whose parents did not report the use of fluoride‐containing products showed a significantly higher quantification of S. dentisani than those who used the products. This finding could indicate an inhibitory effect of fluoride on this bacterium. Few studies have analyzed the effect of fluoride within the diversity of the human oral microbiome, and the results indicate only a minimal effect. However, these studies did not control for the use of water and fluoride products. A study conducted in mice by Yasuda et al., (2017) compared the effects of water and fluoride products and found a selective impact on oral microbiota, especially acidogenic bacteria (Yasuda et al., 2017). Fluoride has been shown to affect the metabolism of S. mutans and Streptococcus sanguis because this ion binds to the active site of the glycolytic enzyme enolase or forms metal‐fluoride complexes (AlF_4_^−^), which inhibit proton translocation by F‐ATPases by limiting the phosphate required in enzymatic reactions. Fluoride also disrupts the ion gradient across the bacterial membrane (Kato, Vasquez, & Moyerbrailean G, 2017). Although S. dentisani is not an acidogenic bacterium, fluoride could have an effect on its metabolism or on tooth adhesion. In 2013, Loskill et al. performed an in vitro study with the bacteria S. mutans, S. oralis, and Staphylococcus carnosus to determine adhesion to hydroxyapatite surfaces treated with a fluoride solution and showed low bacterial adhesion strength by force spectroscopy (Loskill et al., 2013).

In conclusion, the presence of S. dentisani was identified and quantified using the qPCR technique in dental plaque samples of children from four Colombian cities. The study found no significant differences in the quantification of bacteria in caries‐free children with respect to those classified with some caries index level. This could be due to the large variability in S. dentisani levels with a limited sample size or to a lack of association with the oral health parameters studied. Regarding other variables studied in the quantification of this bacterium, a possible relationship was found between the use of fluoride‐containing products, origin, and the frequency in daily food intake. In the future, a potential relationship between the different factors studied in the present work with other potentially beneficial (e.g., ammonia producers) or cariogenic (e.g., acid producers) organisms should be investigated.

The design of new in vitro or in vivo experiments to confirm the possible relationships between S. dentisani and variables such as the use of fluoride‐containing products and the frequencies of food intake could determine the ideal conditions for using this bacterium as a probiotic. Additionally, understanding this relationship would add knowledge of the behavior of this bacterium as part of the oral microbiome and its possible interactions in the oral cavity.

## ACKNOWLEDGEMENTS

The authors thank the Comité Nacional para el Desarrollo de la Investigación (CONADI) of the Universidad Cooperative de Colombia for financing the study.

## CONFLICT OF INTEREST

The authors have no conflicts of interest to declare.

## References

[cre2158-bib-0001] Becker, M. R. , Paster, B. J. , Leys, E. J. , Moeschberger, M. L. , Kenyon, S. G. , Galvin, J. L. , … Griffen, A. L. (2002). Molecular analysis of bacterial species associated with childhood caries. Journal of Clinical Microbiology, 40, 1001–1009. 10.1128/JCM.40.3.1001-1009.2002 11880430 PMC120252

[cre2158-bib-0002] Benítez‐Páez, A. , Belda‐Ferre, P. , Simón‐Soro, A. , & Mira, A. (2014). Microbiota diversity and gene expression dynamics in human oral biofilms. BMC Genomics, 15, 311 10.1186/1471-2164-15-311 24767457 PMC4234424

[cre2158-bib-0003] Benn, A. M. L. , Heng, N. C. K. , Broadbent, J. M. , & Thomson, W. M. (2018). Studying the human oral microbiome: Challenges and the evolution of solutions. Australian Dental Journal, 63, 14–24. 10.1111/adj.12565 28853139

[cre2158-bib-0004] Burton, J. P. , Drummond, B. K. , Chilcott, C. N. , Tagg, J. R. , Thomson, W. M. , Hale, J. D. F. , & Wescombe, P. A. (2013). Influence of the probiotic *Streptococcus salivarius* strain M18 on indices of dental health in children: A randomized double‐blind, placebo‐controlled trial. Journal of Medical Microbiology, 62, 875–884. 10.1099/jmm.0.056663-0 23449874

[cre2158-bib-0005] Camelo‐Castillo, A. , Benítez‐Páez, A. , Belda‐Ferre, P. , Cabrera‐Rubio, R. , & Mira, A. (2014). *Streptococcus dentisani* sp. nov., a novel member of the mitis group. International Journal of Systematic and Evolutionary Microbiology, 64, 60–65. 10.1099/ijs.0.054098-0 24006481

[cre2158-bib-0006] Huang, X. , Palmer, S. R. , Ahn, S. J. , Richards, V. P. , Williams, M. L. , Nascimento, M. M. , & Burne, R. A. (2016). A highly arginolytic Streptococcus species that potently antagonizes *Streptococcus mutans* . Applied and Environmental Microbiology, 82, 2187–2201. 10.1128/AEM.03887-15 26826230 PMC4807514

[cre2158-bib-0007] Jiang, S. , Gao, X. , Jin, L. , & Lo, E. C. (2016). Salivary microbiome diversity in caries‐free and caries‐affected children. International Journal of Molecular Sciences, 17, E1978. 27898021 10.3390/ijms17121978 PMC5187778

[cre2158-bib-0008] Kato, I. , Vasquez, A. , & Moyerbrailean, G. (2017). Nutritional correlates of human oral microbiome. Journal of the American College of Nutrition, 36, 88–98. 10.1080/07315724.2016.1185386 27797671 PMC5477991

[cre2158-bib-0009] Kilian, M. , Chapple, I. L. C. , Hannig, M. , Marsh, P. D. , Meuric, V. , Pedersen, A. M. L. , … Zaura, E. (2016). The oral microbiome—An update for oral healthcare professionals. British Dental Journal, 221, 657–666. 10.1038/sj.bdj.2016.865 27857087

[cre2158-bib-0010] Kralik, P. , & Ricchi, M. (2017). A basic guide to real time PCR in microbial diagnostics: Definitions, parameters, and everything. Frontiers in Microbiology, 8, 108. 28210243 10.3389/fmicb.2017.00108 PMC5288344

[cre2158-bib-0011] Kressirer, C. A. , Smith, D. J. , King, W. F. , Dobeck, J. M. , Starr, J. R. , & Tanner, A. C. R. (2017). *Scardovia wiggsiae* and its potential role as a caries pathogen. Journal of Oral Biosciences, 59, 135–141. 10.1016/j.job.2017.05.002 29104444 PMC5665406

[cre2158-bib-0012] Kreth, J. , Merritt, J. , & Qi, F. (2009). Bacterial and host interactions of oral streptococci. DNA and Cell Biology, 28, 397–403. 10.1089/dna.2009.0868 19435424 PMC2903342

[cre2158-bib-0013] Lee, P. P. , Mak, W. Y. , & Newsome, P. (2004). The aetiology and treatment of oral halitosis: An update. Hong Kong Medical Journal, 10, 414–418. 15591601

[cre2158-bib-0014] López‐López, A. , Camelo‐Castillo, A. , Ferrer, M. D. , Simon‐Soro, A. , & Mira, A. (2017). Health‐associated niche inhabitants as oral probiotics: The case of *Streptococcus dentisani* . Frontiers in Microbiology, 8, 379. 28344574 10.3389/fmicb.2017.00379 PMC5344910

[cre2158-bib-0015] Loskill, P. , Zeitz, C. , Grandthyll, S. , Thewes, N. , Müller, F. , Bischoff, M. , … Jacobs, K. (2013). Reduced adhesion of oral bacteria on hydroxyapatite by fluoride treatment. Langmuir, 29, 5528–5533. 10.1021/la4008558 23556545

[cre2158-bib-0016] Mineoka, T. , Awano, S. , Rikimaru, T. , Kurata, H. , Yoshida, A. , Ansai, T. , & Takehara, T. (2008). Site‐specific development of periodontal disease is associated with increased levels of *Porphyromonas gingivalis, Treponema denticola*, and *Tannerella forsythia* in subgingival plaque. Journal of Periodontology, 79, 670–676. 10.1902/jop.2008.070398 18380560

[cre2158-bib-0017] Mombelli, A. , Van Oosten, M. A. C. , Schürch, E. , & Lang, N. P. (1987). The microbiota associated with successful or failing osseointegrated titanium implants. Oral Microbiology and Immunology, 2, 145–151. 3507627 10.1111/j.1399-302x.1987.tb00298.x

[cre2158-bib-0018] Moynihan, P. J. (2005). The role of diet and nutrition in the etiology and prevention of oral diseases. Bulletin of the World Health Organization, 83, 694–699. 16211161 PMC2626331

[cre2158-bib-0019] Nascimiento, M. M. , Gordan, V. V. , Garvan, C. W. , Browngardt, C. M. , & Burne, R. A. (2009). Correlations of oral bacterial arginine and urea catabolism with caries experience. Oral Microbiology and Immunology, 24, 89–95. 10.1111/j.1399-302X.2008.00477.x 19239634 PMC2742966

[cre2158-bib-0020] Nasidze, I. , Li, J. , Schroeder, R. , Creasey, J. L. , Li, M. , & Stoneking, M. (2011). High diversity of the saliva microbiome in Batwa Pygmies. PLoS One, 6, 23352. 10.1371/journal.pone.0023352 PMC3156759 21858083

[cre2158-bib-0021] Rosier, B. T. , Marsh, P. D. , & Mira, A. (2018). Resilience of the oral microbiota in health: Mechanisms that prevent dysbiosis. Journal of Dental Research, 97, 371–380. 10.1177/0022034517742139 29195050

[cre2158-bib-0022] Sampaio‐Maia, B. , & Monteiro‐Silva, F. (2014). Acquisition and maturation of oral microbiome throughout childhood: An update. Dental Research Journal, 11, 291–301. 25097637 PMC4119360

[cre2158-bib-0023] Simón‐Soro, A. , Guillen‐Navarro, M. , & Mira, A. (2014). Metatranscriptomics reveals overall active bacterial composition in caries lesions. Journal of Oral Microbiology, 6, 25443 10.3402/jom.v6.25443 25626770 PMC4247497

[cre2158-bib-0024] Simón‐Soro, A. , & Mira, A. (2015). Solving the etiology of dental caries. Trends in Microbiology, 23, 76–82. 10.1016/j.tim.2014.10.010 25435135

[cre2158-bib-0025] Wake, N. , Asahi, Y. , Noiri, Y. , et al. (2016). Temporal dynamics of bacterial microbiota in the human oral cavity determined using an in situ model of dental biofilms. NPJ Biofilms Microbiomes, 10, 16018. 10.1038/npjbiofilms.2016.18 PMC5515266 28721251

[cre2158-bib-0026] Yasuda, K. , Hsu, T. , Gallini, C. A. , et al. (2017). Fluoride depletes acidogenic taxa in oral but not gut microbial communities in mice. mSystems, 2, e00047–e00017. 28808691 10.1128/mSystems.00047-17 PMC5547758

[cre2158-bib-0027] Zaura, E. , Nicu, E. A. , Krom, B. P. , & Keijser, B. J. F. (2014). Acquiring and maintaining a normal oral microbiome: Current perspective. Frontiers in Cellular and Infection Microbiology, 4, 85. 25019064 10.3389/fcimb.2014.00085 PMC4071637

[cre2158-bib-0028] Zhang, Y. , Zhen, M. , Zhan, Y. , Song, Y. , Zhang, Q. , & Wang, J. (2017). Population‐genomic insights into variation in *Prevotella intermedia* and *Prevotella nigrescens* isolates and its association with periodontal disease. Frontiers in Cellular and Infection Microbiology, 7, 409 10.3389/fcimb.2017.00409 28983469 PMC5613308

